# The Radiolabeling of a Gly-Sar Dipeptide Derivative with Flourine-18 and Its Use as a Potential Peptide Transporter PET Imaging Agent

**DOI:** 10.3390/molecules25030643

**Published:** 2020-02-02

**Authors:** Andrei Molotkov, John W. Castrillon, Sreevidya Santha, Paul E. Harris, David K. Leung, Akiva Mintz, Patrick Carberry

**Affiliations:** 1 Department of Radiology, Columbia University Medical Center, 722 W. 168th St., Room B05, New York, NY 10032, USA; am3355@cumc.columbia.edu (A.M.); jc944@cumc.columbia.edu (J.W.C.); sreevidya.gp@gmail.com (S.S.); davidleungmd@gmail.com (D.K.L.); 2 Medicine Endocrinology, Columbia University Medical Center, 722 W. 168th St., Room B05, New York, NY 10032, USA; peh1@cumc.columbia.edu

**Keywords:** fluorine-18, peptide transporters, positron emission tomography, radiotracer

## Abstract

We have developed a novel fluorine-18 radiotracer, dipeptide **1**, radiolabeled in two steps from mesylate **3**. The initial radiolabeling is achieved in a short reaction time (10 min) and purified through solid-phase extraction (SPE) with modest radiochemical yields (rcy = 10 ± 2%, *n* = 5) in excellent radiochemical purity (rcp > 99%, *n* = 5). The de-protection of the *tert*-butyloxycarbonyl (Boc) and trityl group was achieved with mild heating under acidic conditions to provide ^18^F-tagged dipeptide **1**. Preliminary analysis of ^18^F-dipeptide **1** was performed to confirm uptake by peptide transporters (PepTs) in human pancreatic carcinoma cell lines Panc1, BxPC3, and ASpc1, which are reported to express the peptide transporter 1 (PepT1). Furthermore, we confirmed in vivo uptake of ^18^F-dipeptide tracer **1** using microPET/CT in mice harboring subcutaneous flank Panc1, BxPC3, and Aspc1 tumors. In conclusion, we have established the radiolabeling of dipeptide **1** with fluoride-18, and demonstrated its potential as an imaging agent which may have clinical applications for the diagnosis of pancreatic carcinomas.

## 1. Introduction

The proton-coupled oligopeptide transport (POT), also called the peptide transport (PTR), comprises a family of transporters which are found in animal, plant, yeast, archaea, and both Gram-negative and Gram-positive bacterial cells [1,2,3]. In mammalian cells, the POT family is comprised of four members (PepT1, PepT2, PHT1, and PHT2) [4,5]. These elements are responsible for coordinating the intracellular transport of small peptides across membranes by coupling to an inwardly directed proton gradient and negative trans-membrane electrical potential.

In humans, the normal tissue distribution of PepT1 is predominantly in the apical plasma membrane of enterocytes in the small intestine where it helps in the absorption of nutrients and small peptides [6,7]. It is also found in renal proximal tubular cells and in bile duct epithelial cells. PepT2 is found in epithelial cells of the kidney tubule, lung, and cerebral cortex. PepT2 function is the reabsorption of di/tri-peptides and peptide-like drugs from the glomerular filtrate [8]. The expression of PepT1 and PepT2 has also been studied in tumor cells [9]. Immunohistochemistry, Western blotting, and gene expression studies revealed that PepT1 and PepT2 are over-expressed and regulated by different kinases in a variety of human cancer cell lines like bile duct epithelial cells and colon, prostate, and pancreatic cancer cell lines [10,11,12,13]. These results suggest that PepT1 and PepT2 may be clinically useful to target as biomarkers of specific epithelial cancers. H^+^/Peptide transporters have been shown to be responsible for peptide transport activity in cancer cells, including pancreatic carcinoma [9,11,14].

**Figure 1:**
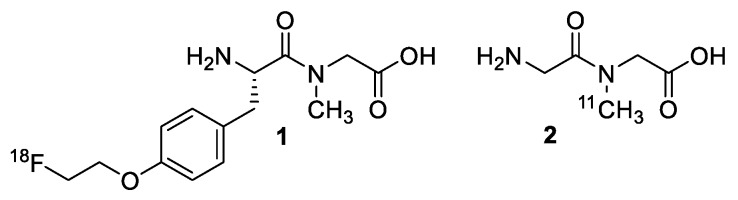
Structure of [^18^F]FEPPG **1** and previously reported [^11^C]Gly-Sar **2**.

The dipeptide PET radiotracer, [^11^C]glycylsarcosine **2** (Gly-Sar) [15] has been shown to be specific in the imaging of PepT2, with a higher specificity to distinguish between tumors versus inflammatory tissue with respect to [^18^F]FDG [9,11]. The design of our dipeptide is a modification from the work performed by Nabulsi et al. [15], in which the dipeptide Gly-Sar was radiolabeled with carbon-11 on the internal amine. We propose here the incorporation of fluorine-18 into a dipeptide Gly-Sar derivative. Brandsch et al. [16] lists criteria that are essential for dipeptides high-affinity substrate-receptor binding for PepT1 and PepT2. Following these suggestions, we designed a dipeptide that possesses a free amine and carboxyl-terminal, a bulky peptide side chain, and an overall hydrophobicity that follows the criteria to increase PepTs binding (Figure 1).

Herein we report the synthesis of a novel ^18^F-dipeptide **1**, (S)-*N*-(2-amino-3-(4-(2-(fluoro-^18^F)ethoxy)phenyl)propanoyl)-*N*-methylglycine ([^18^F]FEPPG), that meets the criteria as a suitable PepT binding substrate. We also demonstrate preliminary data confirming cell uptake of the radiolabeled dipeptide by several human pancreatic carcinoma cell lines and uptake of the [^18^F]FEPPG **1** by μ-PET imaging on subcutaneous human xenograft Panc1, BxPC3, and ASpc1 tumor models in mice. This suggests that [^18^F]FEPPG **1** is suitable for further study to explore its specificity as a PET tracer.

## 2. Results and Discussion

### 2.1. Gly-Sar Dipeptide Derivative Radiolabeled with Fluorine-18

In previous studies [^11^C]Gly-Sar (Figure 1) has been reported [17] as a PET radiotracer for mouse heterotopic pancreatic cancer. It was suggested that [^11^C]Gly-Sar had an advantage over [^18^F]FDG by not binding to inflammation sites allowing to differentiate between inflammatory response and tumor. We propose the use of fluorine-18 nuclide to radiolabel a Gly-Sar dipeptide derivative since fluorine-18 has several important advantages over carbon-11 labeled compounds. Common advantages of fluorine-18 include a relatively high amount of theoretical molar activity (1.71 × 10^3^ Ci/µmol), high resolution with a low positron energy (0.64 MeV), and a half-life of 109.8 min that allows for easier commercial distribution and longer scan times as compared to carbon-11. [18,19]. 

The synthesis of dipeptide **1** was a two-step procedure in which the fluorine-18 was first incorporated in the molecule, followed by the deprotection of the *tert*-butyloxycarbonyl (Boc) and *tert*-butyl group. Initially, solvents were screened (dimethyl sulfoxide, dimethylformamide, and acetonitrile) at a constant temperature, time and concentration to determine the suitable reaction media. From these tests (data not shown), deprotection performed in dimethyl sulfoxide result in least amount of side products, in the highest overall yield; it was therefore used as the solvent in all other subsequent reactions for the optimization of intermediate **4**. Next, a series of temperatures were studied, with 110 °C leading to the highest overall yield with the use of dimethyl sulfoxide as the solvent. 

The optimization of the first step was further evaluated by adjusting the amount mesylate precursor, compound **3**, used for the formation of intermediate **4** (Table 1).

An aliquot (10 µL) of reaction media was removed after 10 min and quenched in 1 mL of 50/50 (*v*/*v*) water/acetonitrile. This sample was evaluated by high-performance liquid chromatography (HPLC) to determine the amount of product formed relative to unreactive fluoride-18 complex and other impurities identified by the radiation detector. The use of 2.0 mg of compound **3** (entry 3, Table 1) provided radiolabeling of intermediate **4** in a 23% conversion. Doubling the amount of precursor from 1.0 mg (entry 2, Table 1) to 2.0 mg led to a 50% increase in the overall conversion of intermediate **4**. However, when 4.0 mg of precursor **3** (entry 3, Table 1) was used, the conversion to radiolabeled compound **4** had no significant increase in yield.

With the amount of starting material, solvent, and temperature determined, our attention turned to optimizing the reaction time for the radiolabeling of intermediate **4**. For this study, the initial labeling reaction was set-up and monitored using an analytical HPLC system. Aliquots were removed at set time points (Table 2) and the formation of desired radiolabeled species **4** was determined relative to other peaks observed in the radio-trace. As shown in Table 2, a 19% conversion of the desired product was attained in 10 min (entry 4). Since additional impurities were detected after 20 min in the radio-trace, 10 min was used as the optimized time for the reaction of mesylate **3** with dry flouride-18 complex.

For purification of ^18^F-intermediate **4**, we tested HPLC with fraction collection as well as solid-phase extraction (SPE) cartridges. We found no detectable differences from the overall decay corrected yield or radiochemical purity achieved with the use of either purification method (data not shown). However, an important advantage of the SPE (t-C18) cartridge was the shortening of the synthesis time, by approximately 30 min, making it an optimal choice.

**Scheme 1:**
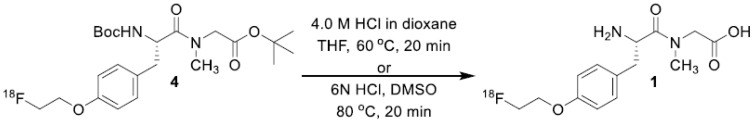
Formation of desired [^18^F]FEPPG **1**.

To obtain the de-protection of both the Boc and *tert*-butyl group in one-step, we screened several reaction conditions. We found that using hydrogen chloride solution (4.0 M in dioxane) and tetrahydrofuran with mild heating (see Method A) led to the final production of [^18^F]FEPPG **1** with the minimal side products [20]. The use of 6 N hydrochloric acid in dimethyl sulfoxide (see Method B) also provided the desired radioligand (Scheme 1). When trifluoroacetic acid or higher temperatures (hydrochloric acid, 90 °C) was used for the de-protection, a partial decomposition of product was observed or sluggish reactions conditions resulted in elongated reaction times with only mono-de-protection of the final product. 

**Figure 2:**
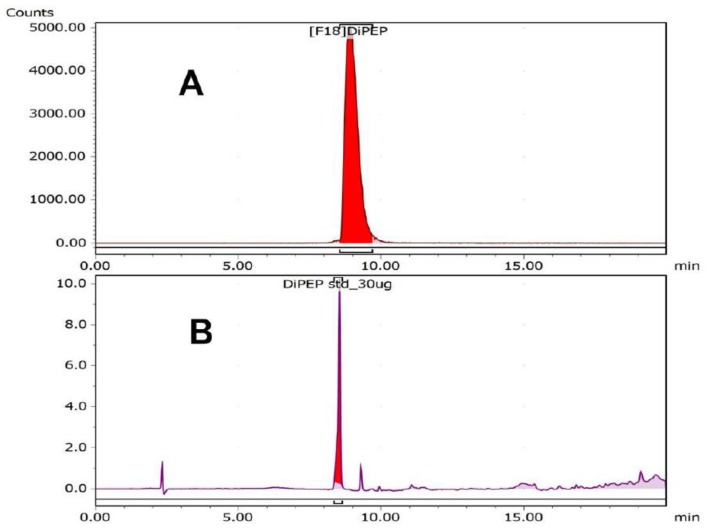
HPLC chromatogram of [^18^F]FEPPG **1**. (**A**) Radiotrace; (**B**) UV @ 280 nm. HPLC conditions: acetonitrile: 0.1% trifluoroacetic acid (aq) solution 10/90 ramp to 90/10 over 20 min; flow rate = 1.5 mL/min; Luna 5 u C18(2) 100 A, 250 × 4.60 nm column Agilent 1260 Infinity (Santa Clara, CA, USA).

Purification of [^18^F]FEPPG **1** was achieved with the use of reverse phase HPLC (Figure 2). The fractions collected representing the desired product were pooled together and assayed to produce approximately 70% decay corrected yield of [^18^F]FEPPG **1**. However, after trapping and releasing (SPE) followed by the formulation process, less than 10% of radioligand was secured in >95% radiochemical purity. Current work is underway with the use of different SPE cartridges. 

The overall decay corrected yield for the formation of [^18^F]FEPPG **1**, starting from a raw solution of aqueous fluoride-18/[^18^O]-enriched water (98%), was 19 ± 9% (*n* = 5) with an average total synthesis time of 117 min. The chemical purity of the final product, determined by HPLC analysis, was 33 ± 5% (*n* = 5) with a range of 0.5–4.8 μg/mL of the fluorine-19 isotope of FEPPG. The radiochemical purity determined by HPLC analysis of [^18^F]FEPPG was determined to be 89 ± 5% (*n* = 5).

### 2.2. Initial Testing of [^18^F]FEPPG in Pancreatic Carcinoma Cell Lines and Tumors

**Figure 3:**
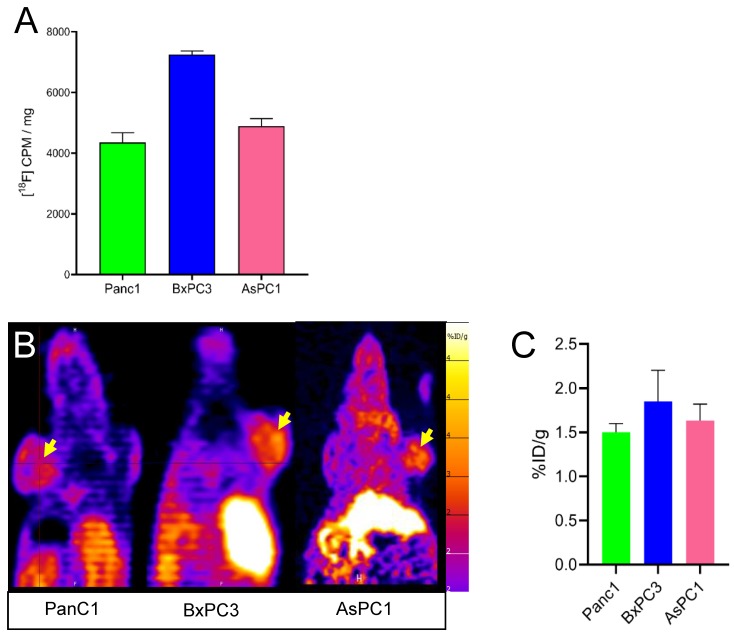
Cell uptake (**A**) and PET scanning (**B**,**C**) using [^18^F]FEPPG **1**. (**A**) Panc1, BxPC3, and AsPC1 human pancreatic carcinoma cell uptake of [^18^F]FEPPG **1**; (**B**,**C**) PET scanning using [^18^F]FEPPG **1** tracer of mice (*n* = 3) with heterotrophic sc tumors originated from Panc1, BxPC3, and AsPC1 cells. Yellow arrow, sq tumor labeled by [^18^F]FEPPG.

While the main goal of this work was to establish the chemical synthesis of [^18^F]FEPPG **1**, we performed initial cell and tumor uptake studies to ensure that newly introduced fluorine-18 moiety did not negate cell and tumor binding previously reported in the carbon-11 analog. We therefore performed initial cell uptake studies on Panc1, BxPC3, and AsPC1 cells, which have been reported to express the PepTs, and found that all tested cell lines had uptake of [^18^F]FEPPG **1** (Figure 3A).

Furthermore, we found similar [^18^F]FEPPG **1** uptake on PET imaging 30 min after injection of 3.7–5.5 MBq (100–150 μCi) of [^18^F]FEPPG **1** (Figure 3B,C) in subcutaneous xenograft models of all pancreatic cancer cell lines tested (Panc1, BxPC3, and AsPC1). As expected, higher amounts of [^18^F]FEPPG **1** uptake were observed in the liver and kidneys, in line with the transporter expression and excretion [21,22,23] (Figure 3B and data not shown). 

In conclusion, we have a feasible synthesis pathway and initial indication of tumor uptake, justifying further study of [^18^F]FEPPG **1**. Our future focus is on examining receptor specificity using unlabeled substrates of PepTs to demonstrate competition as well as defining radioligand binding to specific PEPT transporters by transfecting either PepT1 or PepT2 into non expressing cells and quantitating uptake of [^18^F]FEPPG **1** caused by each transporter. 

## 3. Conclusions

We have reported a new fluorine-18 labeled dipeptide **1**, which is successfully synthesized in two steps. The intermediate was radiolabeled in short reaction time (10 min) and could be easily purified by way of solid-phase extraction (*t-*C_18_ plus cartridge), eluted with acetonitrile, dried, and used directly in the next synthetic sequence without any further purification. The desired final product was formulated into an isotonic injectable solution. Of significance, the final injectable product demonstrated excellent radiochemical purity. We also performed preliminary cell and tumor uptake studies to demonstrate retained in vitro and in vivo uptake of our new molecular entity that has an additional fluorine-18 moiety compared to the originally reported carbon-11 tracer, justifying its further study to confirm PEPT specificity. 

## 4. Material and Methods

### 4.1. General Information

Mesylate precursor **3**, intermediate **4**, and final product standard **1** were purchased from Syngene International Ltd. (Bangalore, India). Hydrogen chloride solution was purchased from Sigma-Aldrich (St. Louis, MO, USA) and used without further purification. Oxygen-18 enriched water (min. 98%) was purchased from Advanced Accelerator Applications (Milburn, NJ, USA) and used for the cyclotron bombardments (2.4 mL per bombardment). The cell lines (AsPC1, Panc1, and BxPC3) and culture media were obtained from American Type Culture Collection (ATCC, Manassas, VA, USA) Company. Fetal bovine serum came from Atlanta Biologicals (Norcross, GA, USA). DMEM was obtained from Fisher Scientific (Pittsburg, PA, USA). Fetal bovine serum (FBS) was obtained from Atlanta Biologicals (Norcross, GA, USA). All other reagents not listed above were of the highest grade available from Sigma-Aldrich (St. Louis, MO, USA) and Fisher Scientific (Pittsburgh, PA, USA). Radiochemical yields (rcy) are reported as decay corrected to the end of cyclotron target bombardment. 

### 4.2. Animals

CrTac:NCr-Foxn1nu mice (Taconic, Rensselaer, NY, USA) referred to in text as NCr were maintained on a normal mouse diet. All animal experiments were conducted according to protocols approved by the Institutional Animal Care and Use Committee of Columbia University Medical Center.

### 4.3. Radiochemistry

#### 4.3.1. (*S*)-*tert*-Butyl 2-(2-((*tert*-butoxycarbonyl)amino)-3-(4-(2-[^18^F]fluoro-ethoxy)phenyl)-*N*-methylpropanamido)acetate (**4**)

A typical procedure for the formation of radioactive intermediate **4** is as follows: [^18^O]-Enriched water (98%) aqueous solution of fluoride-18, produced from a Siemens 111 RDS cyclotron (Knoxville, TN, USA), was trapped on a pre-conditioned QMA light (Waters Corporation, Milford, MA, USA) cartridge. A solution of 1.5 mL (1.0 mL acetonitrile, 0.5 mL water) containing cryptand 222 (15 mg of 4,7,13,16,21,24-hexaoxa-1,10-diazabicyclo[8.8.8]hexacosane) and 3.0 mg potassium carbonate, was passed through the QMA light cartridge into a reaction vessel to provide K[222]^18^F complex. This complex was dried in a heat block at 110 °C with a flow of argon until most of the solvent was evaporated. The solution was further azeotroped with the use of acetonitrile (3 × 1 mL) at 110 °C over a stream of argon gas. A solution of mesylate **3** (2.0 mg, 3.8 µmol) in 0.5 mL of anhydrous dimethyl sulfoxide was added to the “dry” K[222]^18^F complex at 110 °C. After 10 min, the reaction mixture was removed from the heat and allowed to cool. The reaction mixture was then taken up in 20 mL of water and passed through a *t*-C_18_ plus (Waters Corporation) cartridge (activated by flushing with 10 mL ethanol followed by 10 mL water). The cartridge was then washed with another 10 mL of water and eluted into a test tube with 2.0 mL of acetonitrile to provide radiolabeled intermediate **4** (dcy = 10 ± 2%, *n* = 5; rcp > 99%). Radiochemical purity of intermediate **4** was determined by HPLC. HPLC conditions: Luna 5 µ C18(2) 100 Å column, 250 × 4.60 mm, 5 µm; acetonitrile/0.1 M ammonium formate (aq) in 0.5% acetic acid solution 50/50 ramped to 80/20 over 20 min with a flow rate of 2.0 mL/min; r_t_ = 10.05 min. The newly formed intermediate was dried at 90 °C over a stream of argon. The dried compound was taken to the next step.

#### 4.3.2. (*S*)-2-(2-Amino-3-(4-(2-[^18^F]fluoroethoxy)phenyl)-*N*-methylpropanamido)acetic acid, [^18^F]FEPPG (**1**)

**Method A:** A typical procedure for the formation dipeptide **1** is as follows: Tetrahydrofuran (167 µL) followed by hydrogen chloride solution (833 µL, 4.0 M in dioxane) was added to dry intermediate **4**. The reaction mixture was placed in a heat block set to 60 °C. After 20 min, remove from heat and allowed to cool. The reaction mixture was diluted with 9/1 (*v*/*v*) 0.1% trifluoroacetic acid (aq)/acetonitrile (1 mL) solution and purified by HPLC (Luna 10 µ C18(2) 100 Å column, new column 250 × 10 mm; acetonitrile/0.1% trifluoroacetic acid (aq) solution 10/90 ramped to 90/10 over 30 min as mobile phase with a flow rate of 4.0 mL/min; fractions collected from 10.00 to 10.80 min). The fractions were pooled together and diluted with 40 mL of sterile water for injection. The reaction media were then passed through a *t*-C_18_ light (Waters Corporation, Milford, MA, USA) cartridge (activated by flushing with 10 mL ethanol followed by 10 mL water). The *t*-C_18_ light cartridge was then washed with sterile water for injection (10 mL), eluted into sterile 10 mL final product vial with ethanol (0.45 mL) followed by 0.9% saline (2.55 mL). An aliquot was removed for quality control testing.

**Method B:** A typical procedure for the formation of dipeptide **1** is as follows:

Dimethyl sulfoxide (20 µL) followed by 6 N hydrogen chloride (200 µL) was added to intermediate **4**. The reaction mixture was placed in a heat block set to 80 °C. After 20 min, was removed from heat and allowed to cool. The reaction mixture was then taken up in 50 mL of 10% sodium ascorbate solution (pH = 7.5). An aliquot was removed for quality control testing.

Each batch of [^18^F]FEPPG **1** passed all required quality control tests, which included radionuclidic purity (511 keV), chemical (HPLC, identity) and radiochemical purity (HPLC, rcp ≥ 85%), excipients present (acetonitrile < 410 ppm, ethanol < 150,000 ppm), pH (range 4–8), and half-life (110.0 ± 5.0 min). Radiochemical purity/identity of **1** was determined by analytical HPLC. Analytical HPLC conditions: Luna 5 µ C18(2) 100 Å column, 250 × 4.60 mm, 5 µm; acetonitrile/0.1% trifluoroacetic acid (aq) solution 10/90 ramped to 90/10 over 20 min with a flow rate of 1.5 mL/min; r_t_ = 8.68 min. Fluorine-19 derivative of **1** varied from 0.5–4.8 µg/mL, based off calibration curve (y = 1.762x, R^2^ = 0.996, not forced through zero intercept) at 280 nm; molar activity (A_m_) = 17,000 ± 7000 MBq/µmol (460 ± 190 mCi/µmol), where *n* = 5.

### 4.4. Cell Uptake

For cell-uptake studies, Panc1, BxPC3, and AsPC1 human pancreas carcinoma cells were plated at 1 × 10^5^ cells per well in individual 24-well plates 48 h before assay in 10% heat-inactivated FBS in DMEM with 1/100 of anti-anti and 1/200 of glutamine. For non-specific binding control, some wells were left without cells. [^18^F]FEPPG **1**, at ~1.5 MBq per well (~40 μCi), was added to cells for 30 min and washed four times with PBS. Cells were lysed with 0.1 M sodium hydroxide and the resulting gamma-activity was measured on Hidex gamma counter (Hidex, Turku, Finland). For protein concentration, cells were harvested using 100 μL of RIPA buffer; protein concentration was measured using colorimetric assay.

### 4.5. PET Experiments

CrTac:NCr-Foxn1nu mice (Taconic, Rensselaer, NY, USA) were injected sc with 1 × 10^6^ of Panc1, BxPC3 or AsPC1 human pancreatic carcinoma cells (ATCC) in 200 μL of Matrigel^®^. After tumors reached 1 cm^3^, mice were injected IV with 3.7–5.5 MBq (100–150 μCi) of [^18^F]FEPPG **1**. Static 60 min PET was acquired 30 min after [^18^F]FEPPG **1** injection using Inveon micro PET scanner (Siemens, Munich, Germany). PET images were reconstructed using the 3D-OSEM algorithm with three iterations in a 256 × 256 matrix, without attenuation correction (Inveon, Siemens, Munich, Germany) and analyzed using VivoQuant ver 4 (Invicro, Boston, MA, USA).

### 4.6. Quantification and Statistical Analysis

Statistical analysis was performed using Prism 8.0 (GraphPad Software, San Diego, CA, USA). All data are represented as mean ± standard error. Statistical *p*-values were calculated using two-tailed Student’s *t*-test for unpaired samples.

## Figures and Tables

**Table 1:**
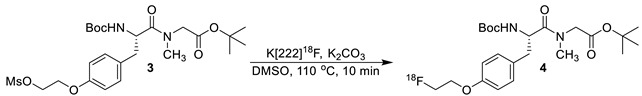
Varying amount of starting material to produce intermediate **4**.

Entry	Amount of 3 (mg)	% Conversion to 4
1	0.5	8
2	1.0	12
3	2.0	23
4	4.0	18

**Table 2:**
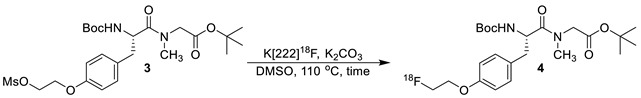
Kinetic study of the labeling of mesylate **3**.

Entry	Time (min)	% Conversion to 4
1	0	ND
2	2	5
3	5	14
4	10	19
5	20	14
6	60	5
